# Integrating an eHealth Program for Pregnant Women in Midwifery Care: A Feasibility Study Among Midwives and Program Users

**DOI:** 10.2196/jmir.988

**Published:** 2009-02-26

**Authors:** Moniek van Zutphen, Ivon E Milder, Wanda J Bemelmans

**Affiliations:** ^1^National Institute of Public Health and the Environment (RIVM)Centre for Prevention and Health Services ResearchBilthovenThe Netherlands

**Keywords:** Internet, health promotion, pregnancy, midwife, implementation, user perception, process evaluation

## Abstract

**Background:**

Health messages may have the highest impact if they are given early in life. Therefore, the Dutch Ministry of Health identified pregnant women as a target population and initiated an innovative eHealth program to serve as a communication channel of health messages to pregnant women.

**Objective:**

The aim was to study the feasibility of implementing this eHealth program into standard midwifery care and to investigate use and user perceptions of the online program among pregnant women.

**Methods:**

All midwifery practices in Amsterdam affiliated with the Dutch Organization of Midwives (n = 25) were requested to implement the eHealth program within a pilot study from March to August 2006. Structured interviews were used to study feasibility of implementation among midwives. During the study period, 488 pregnant women registered themselves on the program website, after which monthly emails were sent to them. The emails were tailored to the stage of pregnancy and provided interactive questions plus answers on six topics and links to related websites. User statistics were registered until January 2007, and user perceptions were assessed with electronic questionnaires.

**Results:**

In total, 80% (20/25) of midwifery practices implemented the program. These midwives gave a short oral explanation about the eHealth program to their clients (n = 1382; about 45% of the total number of clients during this period) and handed out an information brochure. After the pilot, 12 midwifery practices were willing to integrate the eHealth program into their standard care procedures. Regarding program usage, 84% (408/488) of the enrolled women accessed health information within the program. They opened 59% (1296/2213) of the quiz emails and accessed, on average, 16 topics (SD 11). Only 35% (143/408) of users used the hyperlinks to visit related websites. Most women perceived the eHealth information as easy to understand (96%; 157/163) and reliable (81%; 130/161), but only 39% (48/153) agreed that the information was available at the right time. Accessing more topics within the quiz emails was associated with a more positive perception of the program (*P* = .02), but the number of clicks to related websites was not associated with program perception (*P* = .32). The main improvement suggested by program users was to expand the information within the program.

**Conclusions:**

It is feasible to integrate an innovative eHealth program in standard midwifery care, and about half of the practices would like to continue doing so. Program users accessed a substantial proportion of available health information; however, user perceptions were mixed. Therefore, this eHealth program may be a feasible communication channel to promote a healthy lifestyle to pregnant women after suggested revisions have been carried out.

## Introduction

Most women welcome health-related information during their pregnancy and search for pregnancy-related information on the Internet [1-3]. The retrieved information is used, for example, for dietary and other lifestyle changes [4]. This offers an important opportunity to reach pregnant women with relevant health promotion messages that might influence the future health of their child and families. Therefore, the Dutch Ministry of Health initiated an innovative eHealth program to serve as a communication channel of health messages to pregnant women. This program aims to assist women in finding reliable information about a healthy pregnancy and provides links to websites with additional information.

In the Netherlands, 85% of pregnant women start their maternity care at a midwifery practice [5]. Midwives give information about a healthy lifestyle as part of standard care, but this lifestyle-related information is usually confined to the first appointment (at 8-10 weeks of pregnancy). Therefore, the eHealth program is designed to deliver information about a healthy lifestyle throughout the whole period of pregnancy. After enrollment, women receive monthly emails tailored to the stage of their pregnancy until their due date. Since information tailored to the stage of pregnancy is perceived as most useful by pregnant women [3], the eHealth program could be a valuable supplement to usual midwifery care.

We have performed a pilot study among almost 1400 pregnant women to study the feasibility of integrating the eHealth program into standard midwifery care. The process evaluation of this study is carried out in line with the RE-AIM model [6]. This model includes not only dimensions at the individual level but also dimensions that apply to the setting in which research is conducted in the evaluation of the potential public health impact of health promotion interventions. A better understanding of the barriers for implementation of the eHealth program perceived by midwives is of importance to improve the implementation and therefore to increase the public health impact. On the individual level, program usage data and user satisfaction will help to identify which aspects of the program could be improved to increase sustained program use. User satisfaction has been evaluated in previous studies of eHealth programs [7-11]. However, to our knowledge, this is the first study that incorporates factors related to the settings in which an eHealth program is to be implemented (the midwifery practices).

Therefore, the purposes of this paper are (1) to study the feasibility of implementing an online healthy lifestyle program into standard midwifery care, including midwife perceptions of the program, and (2) to analyze use and user perceptions of the eHealth program among pregnant women. The results of both objectives served to further improve the program before its countrywide implementation.

## Methods

### Description of the eHealth Program

The program consisted of monthly emails containing a link to a quiz, with a maximum of six questions, tailored to the stage of pregnancy. The quizzes were stored on a website. On the program website women could register for the program and find more information about it. However, health-related information was only accessible through the emails. The content of the intervention program was especially developed for women with a low education level, by presenting the information in plain language and short text blocks, thus requiring only basic literacy skills. Five health-promoting institutes were responsible for the content of the topic of their relative expertise (nutrition, exercise, lifestyle, smoking, or safety), and the Dutch Organization of Midwives was responsible for the content of the pregnancy topic. In this way, existing pregnancy-related information was brought together within one program.

Each email was personalized with the first name of the participant and the number of weeks she was pregnant, and it contained one question from the quiz. The participant was invited to click on the specified link to find out the answer to that question. Clicking on this link automatically opened a new screen with the quiz.

**Figure 1:**
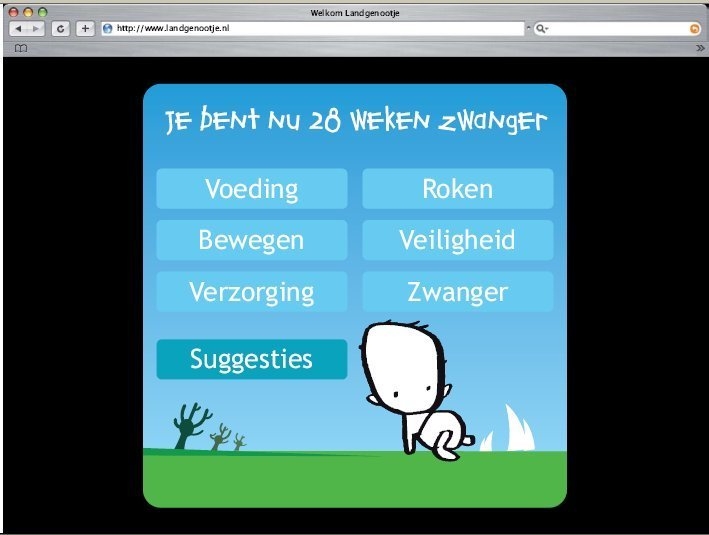
Screenshot of the quiz

After opening the quiz screen, the six topics were displayed (Figure 1). Going over a topic with the mouse displayed the accompanying question. For example, the question at 16 weeks pregnancy about nutrition was “Should you eat more during pregnancy?” and the question at 36 weeks pregnancy about smoking was “Is it still useful to stop smoking?” After clicking on a topic/question, the participant was automatically transferred to the next screen in which the question was shown again together with two possible answers. Selecting an answer was followed by feedback. In this screen, the feedback was followed by a link to additional practical information. An example of practical information concerning lifestyle was “Do you like to drink wine during dinner? Try an alcohol-free wine sometime.” Additionally, hyperlinks to related websites (mainly of the health-promoting institutes) were provided after each topic, so participants could use them to search for additional information.

### Design of Pilot Study

In the Netherlands, midwives are independent paramedical practitioners, qualified to provide full maternity care to women whose pregnancy and childbirth are uncomplicated. The primary task of the Dutch midwife is to monitor the health status of pregnant women and their unborn children. Because of their frequent contacts throughout pregnancy and because of their expertise, midwives are considered to be important information providers for pregnant women, especially for women pregnant with their first child. Therefore, midwifery practices are a unique platform to inform pregnant women about the existence of an eHealth program with relevant and reliable pregnancy-related information. Furthermore, it is likely that information about the eHealth program could easily be incorporated into standard care since general health and lifestyle information is already given during the first midwifery visit.

All 25 midwifery practices in Amsterdam affiliated with the Dutch Organization of Midwives were requested to implement the eHealth program from March to August 2006. They were instructed to give oral information about the program and hand out an information brochure to all of their clients before the 16th week of pregnancy in order to motivate their clients to enroll in the eHealth program. The midwives informed 1382 women about the eHealth program, and 238 women (17%) subsequently registered themselves (at home) on the program website, as described in more detail elsewhere [12]. In addition, 250 women who heard of the program through other channels, such as friends or media, enrolled in the program. So, in total, there were 488 participants.

### Feasibility of Implementation and Midwife Perceptions

In July 2006, the 25 midwifery practices were contacted by telephone to make an appointment for an interview. At this stage we checked if the practice had implemented the eHealth program. Practices that did not (actively) implement the program were asked a few short questions to discover why they did not participate. For the participating practices, a date was set for a structured interview. Preferably, the interview was conducted face-to-face within the midwifery practice with one or more midwives; when this was not possible, the interview was done by telephone. One midwifery practice was unavailable for the interview due to time restraints and personal circumstances of the midwives. However, the practice assistant gave a general impression of the implementation. The questions within the interview were categorized into four sections: implementation, program perception by midwives, points for improvement, and some general information about the midwifery practice. It took approximately 15-30 minutes to complete the interview. The answers of the midwives were summarized by the interviewer and manually recorded during the interview. To estimate the delivery rate of the program within the midwifery practices, the midwives were asked to record each client who was informed about the program on a specifically designed card. Furthermore, during the interview, the midwife gave an estimate of the number of clients visiting the practice.

### Program Use

User statistics were registered until January 7, 2007, by a third party specialized in Internet technology (OOiP, The Netherlands) and included enrollment data, emails sent, quiz questions accessed, practical tips accessed, and related websites visited through hyperlinks within the program. A quiz email was considered as opened if at least one question was accessed. A self-completed questionnaire was used to assess participant characteristics at baseline.

### User Perceptions

**Figure 2:**
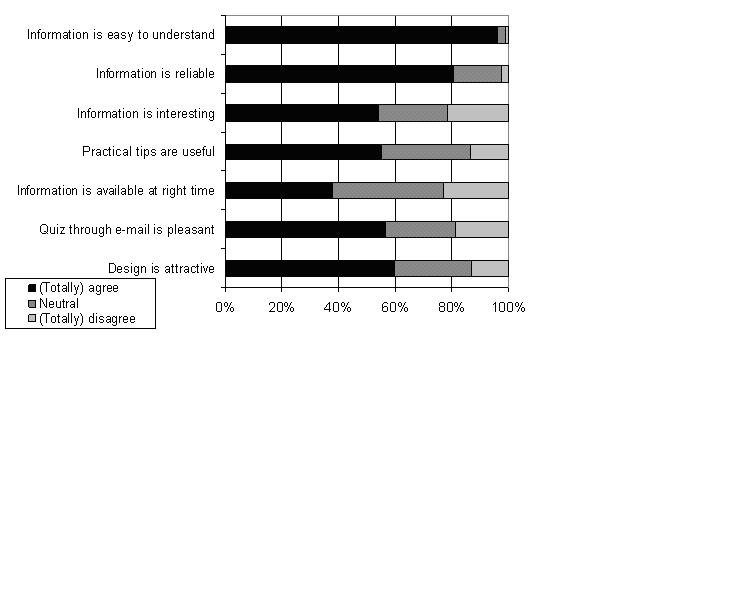
User perceptions of the eHealth program (n = 163)

An online self-administered questionnaire was used to measure user satisfaction with the eHealth program. Invitations to complete the questionnaires were sent by email the week after the participants received the third quiz email, but no invitations were sent after the 40th week of pregnancy. The feedback questionnaire contained several statements about the program that could be rated on a five-point scale (“totally agree” to “totally disagree”). Participants could also indicate which of the six lifestyle topics they enjoyed most and found most useful, and overall satisfaction was rated with a score between 1 (“very bad”) and 10 (“very good”). The questionnaire ended with an open-ended question (“What could we do to improve the program?”). The participants who gave feedback were divided into three groups based on their scores on five user perceptions of the program: interest, usefulness, timing, pleasantness, attractiveness (see last five statements in Figure 2). Those who agreed with four or five statements were classified as having high satisfaction, with two or three statements as having intermediate satisfaction, and with zero or one statement as having low satisfaction.

### Data Analyses

Upon enrollment, a unique personal identification number (ID) was assigned to each program user. The data from the baseline and feedback questionnaires were manually linked to an ID by means of name, zip code, and email address.

Descriptive analyses using means, standard deviations, medians, and percentages were used to describe program use and user attitudes. Chi-square tests were used to detect associations between participant characteristics and user attitudes. Analysis of variance (ANOVA) with follow-up trend analyses was used to detect associations between user satisfaction and program use. Furthermore, linear regression analyses were used to examine the association between five individual perception components (interest, usefulness, timing, pleasantness, attractiveness) and program use (dependent variable). SPSS version 12.0.1 (SPSS Inc, Chicago, IL) was used for all analyses, and statistical significance was set at a level of 0.05.

## Results

### Adoption Rate of Practices

In the pilot project, 80% (20/25) of midwifery practices informed their clients about the program. The other practices did not implement the program, mainly because they considered the program unsuitable for the majority of their clients (highly educated or non-Dutch-speaking women). One practice reported time constraints as the main reason.

### Implementation

The midwives in the 20 participating practices informed their clients about the eHealth program during a standard visit. They all handed out an information brochure, and, with the exception of one midwifery practice, they all gave a short oral explanation about the eHealth program (Table 1). This was most often done during the client’s first visit to the midwife (8-10 weeks of pregnancy). Because this visit is usually used to provide lifestyle-related information, the information brochure was sometimes handed out together with other written materials. All midwives reported that the oral explanation only took a few minutes and that 98% (1354/1382) of women took the information home. Approximately 45% of all clients visiting the participating midwifery practices in the study period received information about the eHealth program. The midwives reported several reasons for not informing all their clients: they forgot (it takes time to integrate into routine care), they did not hand it out to clients without a computer, or the program was judged to be unsuitable for some of their clients (eg, highly educated clients or clients not proficient in Dutch).

Suggestions to facilitate further implementation of the program were to use more promotion materials and to reconsider the moment most appropriate for giving information about the eHealth program.

### Attitudes of Midwives

About half of midwifery practices (12/25) wanted to integrate the eHealth program into their standard care after the end of the study period. Five practices that did not implement the program during the study period would also not implement it after the study period. The other eight practices would not like to continue implementation and had more negative program perceptions than midwives from practices with a positive intention to continue implementation (Table 1). The majority of midwives from practices with a negative intention judged the information within the program as too simple and of little added value to existing information sources. In general, the midwives of practices with a positive intention thought the program would have positive effects on the knowledge of pregnant women about a healthy pregnancy. They had a positive program perception, although some midwives had concerns about the simplicity of some quiz questions and missed having a function to search within all the available information.

**Table 1:** Practice characteristics, program implementation characteristics, and program perceptions of midwifery practices, stratified by the intention to integrate the eHealth program into standard midwifery care after the study period

	Not Implemented (n = 5)	Negative Intention (n = 8)	Positive Intention (n = 11)^a^
**MidwiferyPractice Characteristics**			
Method of interview			
Face-to-face	0	4	7
Telephone	5	4	4
Estimated births in study period, mean (range)^b^	97 (25-170)	210 (150-335)	137 (20-195)
Client ethnicity^b^			
More Dutch than immigrants	2	5	5
Equal numbers of Dutch and immigrants	1	2	1
More immigrants than Dutch	1	1	4
			
**ImplementationCharacteristics**			
Information brochure handed out together with other information	N/A^c^	2	3
Short explanation of eHealth program	N/A	7	11
			
**ProgramPerceptions**			
Opinion on content	N/A		
Positive		0	6
Neutral		2	1
Negative		4	0
Has not seen content		2	4
Opinion on quiz format	N/A		
Positive		1	6
Neutral		4	5
Negative		3	0
Opinion about additive value	N/A		
Valuable		1	9
Not valuable		7	1
Don’t know		0	1
Perception of effectiveness on knowledge about a healthy pregnancy	N/A		
Effective		2	9
Not effective		3	0
Don’t know		3	2
Opinion about national implementation			
Positive	1	2	11
Neutral	2	2	0
Negative	2	4	0

^a^One practice was not available for interview, but the assistant indicated that the practice continued implementation after the pilot period.

^b^Data for one midwifery practice that did not implement the program are missing.

^c^N/A = not available or not applicable.

### Program Use by Pregnant Women

During the study period, 488 pregnant women registered themselves on the program website, and 16% (80/488) of them enrolled but did not access any information available within the program; therefore, they are excluded from all further analysis. The baseline characteristics of participants are summarized in Table 2.

**Table 2:** Baseline characteristics of participants (n = 343)^a^

	%
**Demographic**	
Age (years), mean ± SD	30 ± 5
High level of education	66
Full-time job (≥ 32 hours per week)	56
Non-Dutch ethnicity	26
First-time pregnancy	65
	
**Lifestyle**	
Overweight before pregnancy (BMI ≥ 25)^b^	19
Smoking during pregnancy	
Yes	3
Until pregnancy was known	14
No	84
Alcohol use during pregnancy	17
No use of folic acid supplements	10
	
**Internet**	
Internet at home	96
Used internet > 1 hour per week	86
Used internet to find pregnancy-related information	87

^a^The total number of participants was 488; response rate for self-completed questionnaire at baseline was 70%.

^b^BMI (body mass index) = weight/height^2^(kg/m^2^)

Program users (n = 408) received a mean of 5.4 (SD 1.6) quiz emails and accessed an average of 3.2 (SD 1.8) of those quizzes during their pregnancy (Table 3). A minority (7%) received less than three quizzes because they enrolled in the program late in their pregnancy. Each quiz consisted of six questions (with the exception of the quiz at 16 weeks of pregnancy, which consisted of four questions). The mean number of quiz questions opened during pregnancy was 15.5 (SD 10.7). Thus, each participant accessed, on average, 16 different health promotion messages. The mean number of practical tips opened was 5.8 (SD 6.2). The participants clicked an average of 2.3 (SD 4.1) embedded links, although 65% (266/408) of participants did not click on any link to visit a related website. Of the participants who did visit a related website, 68% (97/142) visited several websites covering five or six different lifestyle topics. Overall, in terms of participation measures, 59% (1296/2213) of sent quiz emails were opened and, within those quizzes, 85% of questions were accessed, but practical tips (37%) and links to related websites were used less often (12%).

**Table 3:** Health-related information received and accessed by program users (n = 408)

	n	%
**Number of quizmails received**		
1-2	29	7
3-5	142	35
6-7	237	58
Mean (SD), median	5.4 (1.6), 6	
	
**Number of quizmails opened**		
1-2	172	42
3-5	186	46
6-7	50	12
Mean (SD), median	3.2 (1.8), 3	
	
**Number of opened quiz questions**		
1-10	166	41
11-20	110	27
21-30	88	22
31-40	44	11
Mean (SD), median	15.5 (10.7), 13	
	
**Number of opened practical tips**		
None	65	16
1-8	242	59
9-16	75	18
17-33^a^	26	6
Mean (SD), median	5.8 (6.2), 4	
	
**Number of clicks to related websites**		
None	266	65
1-6	112	27
7-14	21	5
15-28	9	2
29-40	0	0
Mean (SD), median	2.3 (4.1), 0	
	
**Number of different lifestyle topics visited through hyperlinks**		
None	266	65
1-2	13	3
3-4	32	8
5-6	97	24
Mean (SD), median	1.7 (2.5), 0	

^a^The maximum number of available tips was 33 because not all questions included practical information.

### User Perceptions

Not all participants received three quiz emails and therefore did not receive an invitation to complete the feedback questionnaire (n = 32). Taking this into account, the response rate for the feedback questionnaire was 43% (163/376). Most women evaluated the program as easy to understand (96%; 157/163) and reliable (81%; 130/161), although only 39% (61/161) agreed with the statement that the information was available at the right time (Figure 2). Overall, the average rating was 6.5 (SD 1.5, range 1-10). According to participants, the most useful subjects were pregnancy (31%; 48/153), nutrition (25%; 38/153), safety (23%; 35/153), and lifestyle (14%; 21/153). Almost half (75/152) of program users perceived pregnancy as the most interesting topic, followed by nutrition (20%; 30/152) and lifestyle (16%; 24/152). About half (87/163) of participants who completed the questionnaire also completed the open-ended feedback question. The most common (70%; 61/87) remarks were that they would like to receive more information, more in-depth information, or more variation of information, or they stated that the program gave them little new information.

### Program Use by User Perceptions

The participants who completed the feedback questionnaire were divided into three satisfaction groups based on their responses to the statements about the program (Table 4). The three groups did not differ in the number of quiz emails received (*P* = .81). A higher satisfaction with the program was associated with accessing more information within the program, although there was no significant association between satisfaction and the number of related websites visited (*P* = .32). There was no association between satisfaction and educational level (*P*= .21) or with any other personal characteristic shown in Table 2 (data not shown).

**Table 4:** Participation measures and program rating by user satisfaction

	Low Satisfaction (n = 49)	Intermediate Satisfaction (n = 54)	High Satisfaction (n = 60)	Linear Trend, *P*
Quiz emails received	5.9 ± 1.2	5.7 ± 1.3	5.9 ± 1.0	.81
Quiz emails opened	3.9 ± 1.5	4.2 ± 1.7	4.5 ± 1.4	.04
Quiz questions opened	19.3 ± 10.0	21.7 ± 9.9	23.5 ± 9.0	.02
Practical tips opened	6.5 ± 5.9	8.2 ± 7.0	9.7 ± 7.4	.02
Clicks to related websites	2.7 ± 4.4	2.9 ± 4.0	3.5 ± 4.9	.32
Overal program rating	5.2 ± 1.4	6.5 ± 1.0	7.6± 0.8	<.001

We used regression analyses to investigate the association between individual perception components and program use and found that higher perceived interest and usefulness were associated with accessing more questions (β = −2.4, *P* = .01; β = −2.2, *P* = .04, respectively) and tips (β = −1.6, *P* = .02; β = −2.3, *P* = .002, respectively). Women who found the program interesting or useful accessed about four more questions or tips than women who did not find the program interesting or useful. A higher perceived attractiveness of design was only associated with accessing more tips (β = −2.3, *P* = .003), with women who found the design most attractive opening about four more tips than women who did not find the design attractive. There were no associations between perception components and the number of emails opened or the number of related websites visited. When the five individual components were included simultaneously into one regression model, no significant associations with the number of questions opened were seen, while attractiveness of design was still associated with the opening of tips (β = −1.7, *P* = .04). Furthermore, all five components were associated with the overall rating.

## Discussion

A unique feature of this study is that the feasibility of an eHealth program was studied among both health professionals who provide the program as well as program users. This pilot study shows that it is feasible to integrate an eHealth program into standard midwifery care. About half of the midwifery practices would like to continue implementation of the program. Program users accessed a substantial proportion of available health information, although user perceptions were mixed. Therefore, this eHealth program may be a feasible communication channel to provide reliable information about a healthy lifestyle to pregnant women.

### Practical Implications

A high proportion (80%) of midwifery practices implemented the program. However, this adoption rate may not be achieved with the countrywide implementation. Some of the midwives were actively involved in the development of the program, and there were personal contacts with the publisher of the online program, which probably increased the adoption rate. However, the study implementation was done in a real-life setting and shows promise for the future countrywide implementation since half of the practices would like to integrate the program in their future standard care. Unwillingness to continue implementation of the program was not related to practical barriers, but to how midwives perceived the program. More practices may integrate the program into their future standard care if it was emphasized that the program is designed to bring existing information together and that this feature helps women to gather all relevant information easily, since several midwives had a negative perception of the program because it contained little information that was not already available from other sources.

The program users accessed, on average, 16 different health promotion messages during their pregnancy, which means that, in general, about half of the available health information was seen. Because user perceptions were mixed and several revisions were suggested, the program will be improved before the countrywide implementation, which may increase program usage.

It is thought that program satisfaction within the study is somewhat overestimated. The women who completed the feedback questionnaire accessed more topics (*P*< .001), tips (*P* < .001), and related websites (*P*= .02) than women who did not complete the questionnaire, and satisfaction was associated with more intensive use of the program. However, women who completed the questionnaire did not differ in baseline characteristics compared to women who did not complete the feedback questionnaire, with the exception of a more favorable smoking status among women who gave feedback. There was no association between education level and satisfaction with the program, although the program was designed specifically for women with a lower education. The frequent remark that the program hardly contained any new information was made by women with both a high and low education.

Our results showed that the perceived attractiveness of design was an important factor influencing program use.

### Strengths and Limitations

A strength of our study is that we studied feasibility of the program among both users and midwives using a combination of quantitative and qualitative data. The user perceptions are supplemented with objective usage data, which provide further insight into the program features that need improvement. Furthermore, the evaluation among midwives indicated that the implementation of the program is feasible in a real-life situation.

In most midwifery practices several midwives work together. A limitation of the study is that the interviewed midwives might have had more positive attitudes toward the program than their non-interviewed colleagues. Therefore, we asked all midwives to register all clients to whom they gave information about the program. These data were used to estimate a more objective program delivery rate. The interviews gave insight into the reasons why midwives did not advocate integration of the program into standard care.

Some midwives expressed concerns about the reach and effectiveness of the program. Indeed, previous work showed that disadvantaged women were least easily reached by the health information available in the eHealth program [12], and whether this program positively influences the future health of the child and family remains to be studied.

### Comparison With Prior Work

To our knowledge, only one other eHealth program that made use of emails to promote health behaviors has been evaluated previously [13]. This program was implemented among employees of a large health insurance company and delivered daily health tips during working days for 26 weeks with embedded links for self-monitoring tools and additional information. Their results showed that approximately 60% of participants opened four or five emails per week for most of the study period. This might imply that the emails to pregnant women could be sent much more frequently than once a month, which is in agreement with our finding that participants would like to receive more information. With regard to embedded links, it was found that approximately 90% of participating employees sought additional information at least once, which is much higher than the 35% in our study. This might simply be due to the higher number of available links in their program (> 250) compared to ours (40) or to other factors, such as a more stimulating description of the content of the related website (eg, “Learn new moves with these fitness videos” compared to “Would you like to know more? Have a look on [site address]”).

To our knowledge, there are no other studies that evaluate the feasibility of integrating an Internet program with health information within standard health care. One study introduced an eHealth program to general practitioners to support their patients with lifestyle changes. However, this study focused on practitioner views toward program usability and program design [14].

### Conclusions

It seems feasible to integrate an eHealth program into standard midwifery care. About half of midwifery practices would like to continue recommending an eHealth program to their pregnant clients, and hardly any practical barriers to implementation were reported. Program users accessed a substantial proportion of available health information; however, user perceptions were mixed. Therefore, this eHealth program may be a feasible communication channel to promote a healthy lifestyle to pregnant women after suggested revisions have been carried out. Further research should evaluate email intervals, user needs, and attractiveness of embedded links to optimize transfer and uptake of information.
